# Transcriptome analysis of human cholangiocytes exposed to carcinogenic 1,2-dichloropropane in the presence of macrophages in vitro

**DOI:** 10.1038/s41598-022-15295-3

**Published:** 2022-07-02

**Authors:** Abigail Ekuban, Shigeyuki Shichino, Cai Zong, Frederick Adams Ekuban, Kazuo Kinoshita, Sahoko Ichihara, Kouji Matsushima, Gaku Ichihara

**Affiliations:** 1 Department of Occupational and Environmental Health, Faculty of Pharmaceutical Sciences, Building No. 15, Tokyo University of Science, 2641 Yamazaki, Noda, Chiba 278-8510 Japan; 2 Division of Molecular Regulation of Inflammatory and Immune Diseases, Research Institute of Biomedical Sciences, Tokyo University of Science, Noda, 278-0022 Japan; 3 Evolutionary Medicine, Shizuoka Graduate University of Public Health, Shizuoka, 420-0881 Japan; 4 Department of Environmental and Preventive Medicine, Jichi Medical University School of Medicine, Shimotsuke, 329-0498 Japan

**Keywords:** Cancer, Chemical biology, Environmental sciences, Oncology

## Abstract

1,2-Dichloropropane (1,2-DCP), a synthetic organic solvent, has been implicated in causality of cholangiocarcinoma (bile duct cancer). 1,2-DCP-induced occupational cholangiocarcinoma show a different carcinogenic process compared to common cholangiocarcinoma, but its mechanism remains elusive. We reported previously that exposure of MMNK-1 cholangiocytes co-cultured with THP-1 macrophages, but not monocultured MMNK-1 cholangiocytes, to 1,2-DCP induced activation-induced cytidine deaminase (AID) expression, DNA damage and ROS production. The aim of this study was to identify relevant biological processes or target genes expressed in response to 1,2-DCP, using an in vitro system where cholangiocytes are co-cultured with macrophages. The co-cultured cells were exposed to 1,2-DCP at 0, 0.1 or 0.4 mM for 24 h, and then the cell lysates were assessed by transcriptome analysis. 1,2-DCP upregulated the expression of base excision repair genes in MMNK-1 cholangiocytes in the co-cultures, whereas it upregulated the expression of cell cycle-related genes in THP-1 macrophages. Activation of the base excision repair pathway might result from the previously observed DNA damage in MMNK-1 cholangiocytes co-cultured with THP-1 macrophages, although involvement of other mechanisms such as DNA replication, cell death or other types of DNA repair was not disproved. Cross talk interactions between cholangiocytes and macrophages leading to DNA damage in the cholangiocytes should be explored.

## Introduction

1,2-Dichloropropane (1,2-DCP) is a synthetic chlorinated organic solvent widely used in the production of other organic solvents and in the offset color-proof printing industry to remove ink from the transcription rubber roller^1^. In Japan, 1,2-DCP was first linked to the development of cholangiocarcinoma in some workers of printing industries exposed to 1,2-DCP^2^. The workers diagnosed with cholangiocarcinoma were young adult males aged 25–45 years (mean 36 years), who were free of common cancer risk factors^3,4^. Cholangiocarcinoma is cancer that arises from biliary epithelium of the liver and the extrahepatic bile ducts. Its risk factors include among others, histories of primary sclerosing cholangitis (PSC), pancreaticobiliary maljunction, and hepatolithiasis^5^. 1,2-DCP-induced cholangiocarcinoma was recognized as an occupational disease by the Ministry of Health, Labor, and Welfare of Japan^6^. The occupational cholangiocarcinoma cases showed early onset, compared to common cholangiocarcinoma^3,7^. In 2017, the International Agency for the Research on Cancer (IARC) reclassified 1,2-DCP from group 3 (not classifiable as to its carcinogenicity to humans) to group 1 (carcinogenic to human)^1^. A study indicated that the carcinogenic process involved in this type of occupational carcinoma was different from that of common cholangiocarcinoma^7^. Histopathological examination of surgically resected specimens of occupational cholangiocarcinoma showed precancerous or early cancerous lesions, such as biliary intraepithelial neoplasia (BilIN) and/or intraductal papillary neoplasm of the bile duct (IPNB)^3,7^. Furthermore, sclerosis of the bile duct with variable inflammatory cell proliferation were observed at various sites of the bile ducts in the noncancerous hepatic tissues^3^.

However, the mechanism of 1,2-DCP-induced cholangiocarcinogenesis remains elusive. 1,2-DCP has been suggested to be activated in the liver by the enzyme cytochrome P450 2E1 in mice^8,9^. It is reported to primarily target cholangiocytes in humans, as patients diagnosed with occupational cholangiocarcinoma had elevated levels of γ-glutamyl transpeptidase (γ-GTP) before elevation of aspartate aminotransferase (AST) or alanine aminotransferase (ALT), suggesting hepatic damage is secondary to development of cholangiocarcinoma^5,10,11^. Moreover, with inhalation, 1,2-DCP reaches the large bile ducts without being metabolized in the liver, as blood supply to the cholangiocytes is mainly from the hepatic artery^11,12^.

Previous mechanistic studies reported that exposure to 1,2-DCP induced the expression of mutagenic enzyme activation-induced cytidine deaminase (AID) in cholangiocytes, in the presence of macrophages^13^. We also reported recently that in vitro exposure of human cholangiocytes to 1,2-DCP increased LDH cytotoxicity, DNA damage and ROS production and these changes occurred only in the presence of macrophages^11,14^. The above studies suggest that intercellular signaling plays a significant role in 1,2-DCP-related cytotoxicity, DNA damage and ROS production in cholangiocytes.

Transcriptomic technology is mainly used to read the entire RNA transcripts at a particular time point, in a given biological sample, and has been employed in several studies to provide a clearer picture of the transcriptional profiles and hence a better understanding of certain diseases or a given outcome^15^.

For a better understanding of the molecular mechanism of 1,2-DCP-induced cholangiocarcinogenesis, we investigated the transcriptomic profiles of MMNK-1 cholangiocytes co-cultured with THP-1 macrophages exposed to 1,2-DCP for 24 h. The results identified the involvement of intracellular mechanisms and possibly intercellular signaling pathways, both in MMNK-1 cholangiocytes and THP-1 macrophages, in 1,2-DCP-induced cytotoxicity and DNA damage in MMNK-1 cells.

## Materials and methods

### Cell lines and cell cultures

MMNK-1 cells (human immortalized cholangiocytes) obtained from the Japan Collection of Research Bioresources Cell Bank (JCRB, Osaka, Japan) were maintained in low-glucose Dulbecco’s Modified Eagle Medium, (DMEM, Wako Pure Chemical Industries, Osaka, Japan) and supplemented with 5% heat inactivated fetal bovine serum (FBS) (lot #S17692S1820, Biowest, Riverside, MO) at 37 °C under 5% CO_2_ atmosphere. The cells were detached with Accutase (Innovative Cell Technologies, San Diego, CA) and passaged every 2–3 days. The MMNK-1 cell line are known to express various cholangiocyte markers (e.g., cytokeratin (CK-7 and CK-19) and exhibit cholangiogenic tubule formation (by matrigel assay)^16^. THP-1 cells (Human monocytic cells) were obtained from American Type Culture Collection (ATCC, Rockville, MD) and maintained in Roswell Park Memorial Institute medium 1640 (RPMI1640, Wako, Japan) supplemented with 10% heat inactivated FBS, penicillin, streptomycin, l-glutamine (Gibco, Thermo Fisher, Waltham, MA), and 2-mercapethanol (0.05 mM, Sigma Aldrich, St. Louis, MO), at 37 °C in an atmosphere of 5% CO_2._ The cells were subcultured every 3–4 days. THP-1 cells express Fc and C3b receptors, and they possess HLA-A2, -A9, -B5, -DRW1 and -DRW2 histocompatibility antigens of human lymphocyte antigen (HLA) typing^17^. THP-1 cells were allowed to differentiate into macrophages by incubation with phorbol 12-myristate 13-acetate (PMA, Sigma-Aldrich) at concentration of 162 nM, over a period of 48 h, at 37 °C under an atmosphere of 5% CO_2_, as described previously^18^.

### Co-culture method

PMA (162 nM)-treated THP-1 cells were seeded at 3 × 10^5^ cells/well, in 6-well cell culture inserts with membrane of pore size 0.4 µm (Corning, Kennebunk, ME) and incubated at 37 °C under an atmosphere of 5% CO_2_ for 48 h. The inserts were then washed three times and incubated for 4–5 h at 37 °C under an atmosphere of 5% CO_2_ in fresh complete medium for THP-1 cells. MMNK-1 cells were seeded at 1.5 × 10^5^ cells/well, in 6-well plates and cultured for 12 h, then co-cultured with 48-h-differentiated THP-1 macrophages, for an additional period of 12 h in a mixture of DMEM and RPMI 1640 (1:1 ratio), supplemented with 5% FBS. This was followed by 1,2-DCP exposure at different concentrations for 24 h, as described previously^13^.

### Determination of 1,2-DCP exposure concentration

The ambient concentration of 1,2-DCP where workers developed cholangiocarcinoma is estimated to range from 100 to 670 ppm^2^. The estimated range of 1,2-DCP exposure levels during the process of ink removal was reported to be 150–620 ppm^19^, which are comparable to occupational exposure levels to other organic solvents in poorly ventilated workplaces, which ranged from several hundreds to 1000 ppm^20,21^. To determine the equivalent 1,2-DCP concentrations to be used in our cell culture studies that match the above blood levels, we used the following assumptions; human blood: air partition coefficient of 10.7^1,22^, and concentration of inhaled 1,2-DCP being 1000 ppm (v/v) (0.22 ppm = 1 mg/m^3^). This implies 1000 ppm (4545 mg/m^3^) of 1,2-DCP vapor, is in equilibrium with approximately 0.4 mM (4545 × 10.7 mg/m^3^ = 48,636/112.98 mol/m^3^ = 431 mol/m^3^ ~ 0.4 mM) of 1,2-DCP in blood^11^. Based on these assumptions, we used 0.1 and 0.4 mM for 1,2-DCP concentrations in the present study, representing comparable levels to those found in workers exposed to 1,2-DCP.

### Preparation of 1,2-DCP

1,2-DCP of 98% purity was purchased from Tokyo Chemical Industry (TCI, Tokyo, Japan) and dissolved in dimethyl sulfoxide (DMSO, Wako, Japan). It was subsequently diluted in complete medium for co-cultures of MMNK-1 and differentiated THP-1 cells. The DMSO concentration in the complete medium was adjusted to 0.1% for both the control group and 1,2-DCP-exposed group.

### Exposure of cells to 1,2-DCP

The seeded cells were exposed to 1,2-DCP at 0, 0.1 or 0.4 mM for 24 h and then incubated at 37 °C, sealed in a Tedlar polyvinyl fluoride (PVF) gas sampling bags, as described in detail previously with minor modification^13^.

### Bulk-RNA sequencing library preparation

Cell culture media were aspirated from the co-cultured cells exposed to 1,2-DCP for 24 h. The cells were then put on ice and washed with ice-cold PBS. The cell lysates were prepared using lysis buffer which comprised of lysis binding buffer [100 mM Tris–HCl, pH 7.5, 500 mM LiCl, 10 mM EDTA, 1% LiDS, 5 mM dithiothreitol (DTT)]. PolyA RNAs were isolated using Dynabeads M-270 Streptavidin (Thermo Fisher Scientific, MA) conjugated with biotin-3′ WTA-EcoP-dT25, reverse-transcribed and amplified according to the previous report with some modifications (GSE110711). Produced cDNA was quantified using Nanodrop (Thermofisher Scientific, Waltham, MA) and the purity was confirmed using Bioanalyzer (Agilent, Santa Clara, CA). 100 ng of the whole-transcriptome library was subjected to fragmentation/end-repair/A-tailing using NEBNext Ultra II FS DNA Library Prep Kit for Illumina (New England Biolabs Inc., Tokyo, Japan) with some modifications. The thermal cycling was performed as following condition: for 20 min at 32 °C, 30 min at 65 °C, and hold at 4 °C. Then, 1.25 μL of 1.5 μM CS1 adapter was used for adapter ligation. Ligated products were purified by double size selection with 0.41 × → 0.31 × (final 0.72 ×) AmPure XP beads and eluted with 10 μL of nuclease-free water. The barcoding PCR was performed with 25 μL of barcoding mix [7.5 μL of the resulted eluates, 1 μM primers (IonA_BC[X]_CS1 and trP1 primers), and 1 × NEBNext Ultra II Q5 (New England Biolabs Inc., Tokyo)], and the thermal cycling was performed as following condition: for 30 s at 98 °C, 9 cycles of 10 s at 98 °C and 75 s at 65 °C, followed by 5 min at 65 °C, and hold at 4 °C. Resultant products were purified twice by double size selection with 0.7 × → 0.7 × (final 1.4 ×) AmPure XP beads and eluted with 12 μL of 10 mM Tris–HCl pH8.0. Size distribution of amplified products was analyzed by MultiNA system (Shimazu, Kyoto, Japan) with appropriate dilutions. Final transcriptome libraries, whose lengths were around 300 base pairs, were quantified using the KAPA Library Quantification Kit (KAPA Biosystems, Wilmington, MA). Pooled libraries were sequenced by using Ion 540 Kit-Chef, Ion 540 Chip kit, and an Ion Genestudio S5 Sequencer (Thermo Fisher Scientific), according to the instructions provided by the manufacturer.

### Transcriptome data analysis

Adapter trimming and quality filtering of sequencing data were performed by using Cutadpat-v2.10. The filtered reads were mapped to reference RNA (GRCh38 release-101) using Bowtie2-2.4.2 (parameters: -p 2 -L 16 --very-sensitive-local -N 1 -nofw -seed 656565 -reorder) and read number of each gene were counted. Transcriptome data analysis was performed according to the previous report^23^. In brief, between-sample normalization was performed against raw count data by using R 3.5.1. (https://cran.r-project.org/) and TCC package (EEE-E method)^24,25^. Transcriptome data of 1,2-DCP-exposed MMNK-1 cholangiocytes and THP-1 macrophages genes, with p-values of less than 0.05, fold change ≥ 1.5, and maximum expression ≥ 30 were identified as statistically significant differentially expressed genes.

### PCA plots and volcano plots

Data normalization and differentially expressed genes (DEGs) identification between samples was performed by TCC package^24^. Then PCA analysis was performed with function “prcomp” in the “stats” package of R software^26^. Volcano plots of DEGs between groups were generated with “EnhancedVolcano” package^27^.

### Detection of co-expressed gene modules

Co-expressed gene modules among differentially expressed genes in 1,2-DCP-exposed MMNK-1 cholangiocytes and THP-1 macrophages were detected using Weighted Gene Co-expression Network Analysis (WGCNA) package^28^ in R 3.5.1. Variance-stabilizing transformation of TCC-normalized count data was performed using the DESeq2 package^29^ in R 3.5.1, and the transformed data were used as input in the WGCNA package. The power value used was 9 for MMNK-1 cholangiocytes and 10 for THP-1 macrophages, while the merge_thre value was 0.2, the threshold value for the output of co-expression interactions was 0.25, and other calculation settings were set to defaults in the WGCNA for both MMNK-1 cholangiocytes and THP-1 macrophages.

The genes in the gene module groups detected by WGCNA were further clustered into positively and negatively correlated gene groups by using pheatmap package in R 3.5.1.

### Enrichment analyses

Gene Ontology (GO) and the Kyoto encyclopedia of genes and genomes (KEGG) pathway analysis of differentially expressed genes, were performed using the free online platform; the WEB-based gene set analysis Toolkit (WebGestalt), accessed at http://www.webgestalt.org^30^. These were used to determine the over-representation (enrichment) analyses of the study with set parameters of a minimum of five genes and maximum of 2000 genes for a category, and False Discovery Rate (FDR) cut-off value of < 0.05, using Benjamini–Hochberg method for multiple test adjustment.

### Selection of genes whose expression was dose-dependently changed by exposure to 1,2-DCP

Independently from cluster analysis and subsequent enrichment analysis, we used the Pearson correlation coefficient to assess the significance of dose-dependent changes in the expression of each gene following exposure to 1,2-DCP. In this analysis, the p-value of the difference in the expression level was adjusted using the Benjamini–Hochberg method^31^ and expressed as q-value.

### Expression of genes selected by hypothesis

In addition to the above comprehensive analysis, hypothesis-driven statistical tests were conducted. Since macrophages play diverse functions in the immune response to foreign substances and toxicants^32^ and our previous studies showed 1,2-DCP-induced upregulation of AID, LDH cytotoxicity, DNA damage and ROS production in MMNK-1 cells only when they were co-cultured with THP-1-derived macrophages^11,13,14^ suggesting involvement of intercellular signals, we conducted one-way analysis of variance (ANOVA) and *post hoc* Dunnett’s multiple comparison for the expression levels of cytokines of TNF superfamily or interleukins, chemokines (*CCL, CXCL, CL* and *CX3CL*), cytokine/chemokine-related proteins and cytokine/chemokine receptors in MMNK-1 cholangiocytes or THP-1 macrophages after 24-h exposure to 1,2-DCP. We also conducted ANOVA and post hoc Dunnett’s multiple comparison for expression levels of genes categorized for KEGG’s base excision repair (BER), homologous recombination (HR) and non-homologous end joining (NHEJ) pathway. ANOVA and *post hoc* Dunnett’s multiple comparison test were conducted using JMP Pro version 16.1.0 (SAS Institute Inc. Cary, NC).

## Results

### Transcriptomic analysis of 1,2-DCP exposed MMNK-1 cholangiocytes and THP-1 macrophages identified 1,2-DCP exposure-associated gene signatures

**Figure 1:**
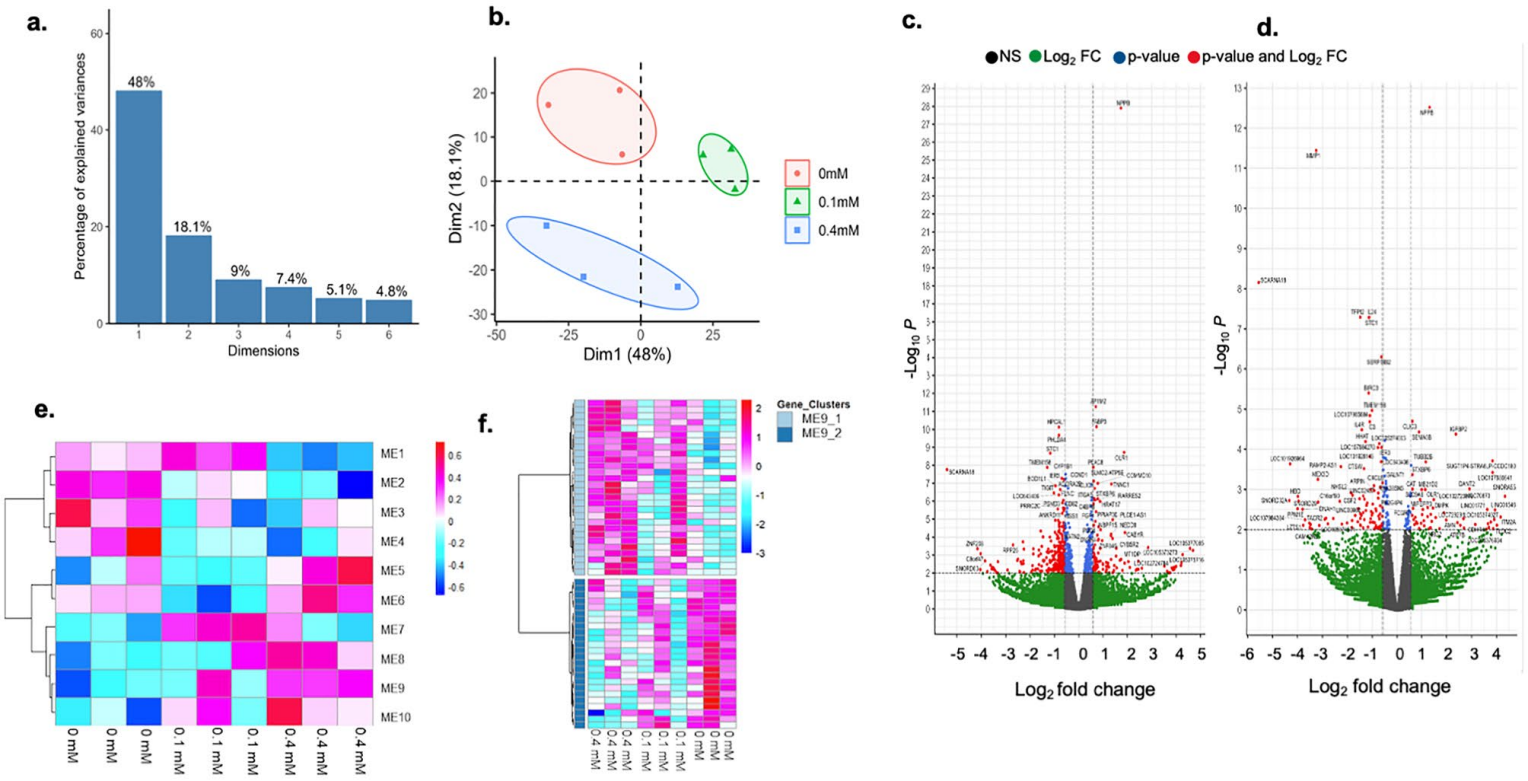
PCA analysis, volcano plot and cluster analysis for identified DEGs in MMNK-1 cholangiocytes co-cultured with THP-1 macrophages when exposed to 1,2-DCP at 0, 0.1 or 0.4 mM for 24 h for 3 independent experiments (n = 3). (**a**) The identified PCA dimensions 1 to 6 and (**b**) PCA Dimension 1 and 2 plotted for each sample; (**c**) screening for DEGs between 0 and 0.1 mM using volcano plot for MMNK-1 cholangiocytes where red-colored dots represent genes of p-value < 0.01 and |Log_2_FoldChange| > 0.58, (**d**) screening for DEGs between 0 and 0.4 mM using volcano plot for co-cultured MMNK-1 cells where red-colored dots represent genes of p-value < 0.01 and |Log_2_FoldChange| > 0.58; (**e**) Heatmap representation of co-expressed gene modules identified by the weighted co-expression network analysis of MMNK-1 cholangiocytes, where each column represents exposure group, whereas each row represents an individual module eigengene. (**f**) Heatmap representation of the module eigengene 9 (ME9) of MMNK-1 cholangiocytes, where each column represents exposure group, whereas each row represents an individual gene.

**Figure 2:**
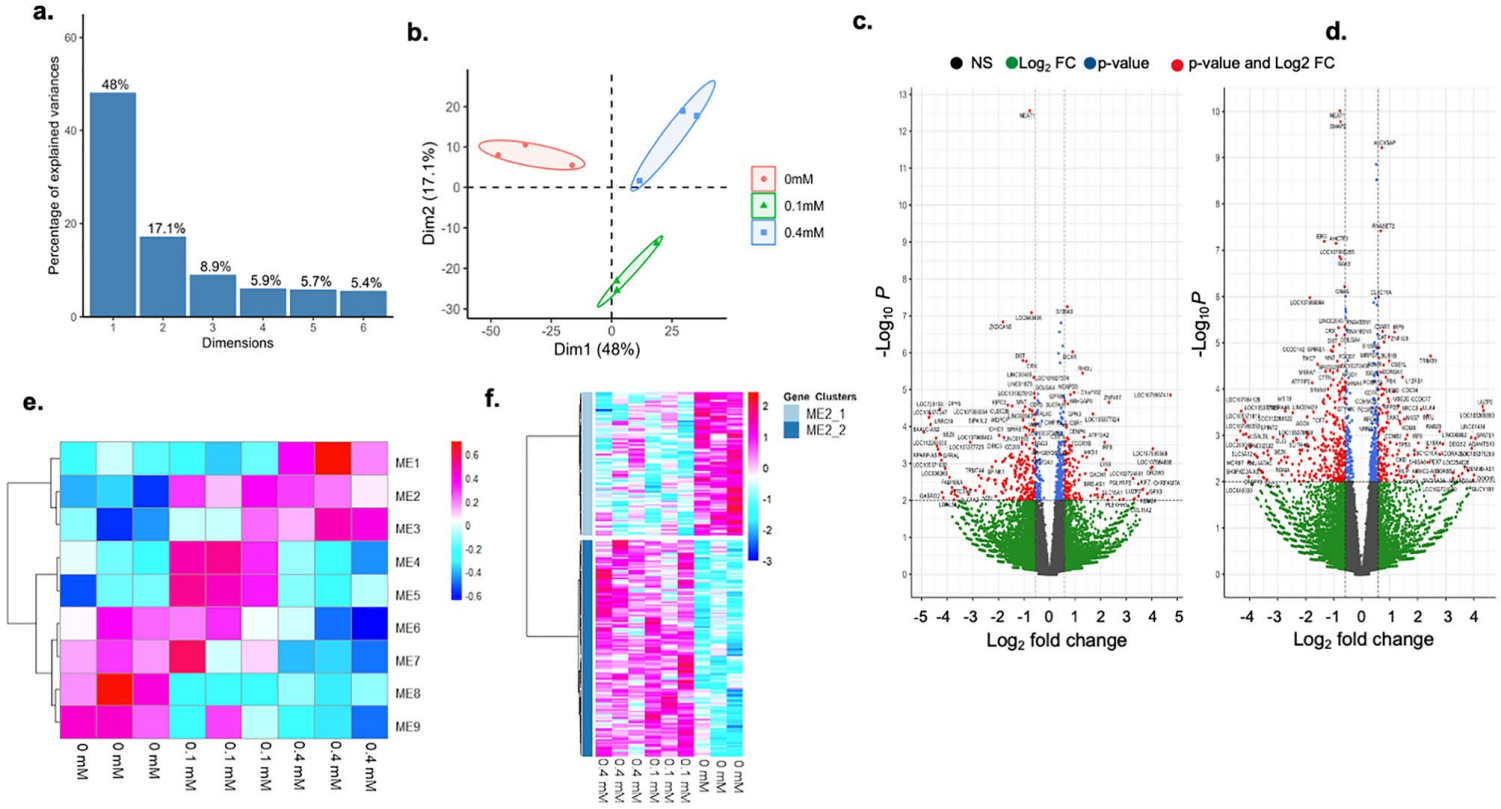
PCA analysis, volcano plot and cluster analysis for identified DEGs of THP-1 macrophages co-cultured with MMNK-1 cholangiocytes when exposed to 1,2-DCP at 0, 0.1 or 0.4 mM for 24 h for 3 independent experiments (n = 3). (**a**) The identified PCA dimensions 1 to 6. (**b**) PCA Dimension 1 and 2 were plotted for each sample. (**c**) screening for DEGs between 0 and 0.1 mM using volcano plot for THP-1 cells where red-colored dots represent genes of p-value < 0.01 and |Log_2_FoldChange| > 0.58, (**d**) screening for DEGs between 0 and 0.4 mM using volcano plot for THP-1 macrophages where red-colored dots represent genes of p-value < 0.01 and |Log_2_FoldChange| > 0.58, (**e**) heatmap representation of co-expressed gene modules identified by the weighted co-expression network analysis of THP-1 macrophages, where each column represents exposure group, whereas each row represents an individual module eigengene, (**f**) heatmap representation of the module eigengene 2 (ME2) of THP-1 macrophages, where each column represents exposure group, whereas each row represents an individual gene.

To identify gene clusters that showed expression changes in line with dose escalation of 1,2-DCP exposure, we first performed transcriptomic analysis of 1,2-DCP exposed MMNK-1 cholangiocytes and THP-1 macrophages. We identified 1,052 and 1525 differentially expressed genes (DEGs) in MMNK-1 cholangiocytes and THP-1 macrophages, respectively (Figs. 1a,b, and 2a,b). PCA analysis showed that the percentages of variance attributed by four dimensions were 48, 18.1, 9 and 7.4%, respectively, and 82.5% in total in MMNK-1 cholangiocytes, and 48, 17.1, 8.9 and 5.9%, respectively, and 79.9% in total in THP-1 macrophages. PCA analysis revealed that 0.1 mM group was more similar to 0 mM group than 0.4 mM group by the component 2 (18.1%) axis in the MMNK-1 cholangiocytes, suggesting that 1,2-DCP dose-dependent gene-expression changes might compose a major part of gene-expression changes in our dataset.

Volcano plots identified the genes in the co-cultured MMNK-1 cholangiocytes between 0 mM group and 0.1 mM group including *NPPB* (p = 7.36E−26), *AP1M2* (p = 4.25E−09)*, FABP3* (p = 6.31E−09)*, OLR1* (p = 2.98E−08) and *PLAC8* (p = 1.37E−07), which were highly significantly or highly upregulated, and then 0 mM group and 0.4 mM group including *NPPB* (p = 7.36E−26)*, CLIC3* (p = 4.26E−07)*, IGFBP2* (p = 8.7E−06)*, SEMA3B* (p = 1.24E−04), and *TUBB2B* (p = 7.16E−04), which were highly significantly or highly upregulated (Fig. 1c,d). In the co-cultured THP-1 macrophages, volcano plots identified genes between 0 mM group and 0.1 mM group including *S100A9* (p = 5.09E−08), *DCXR* (p = 7.8E−06), *RHOU* (p = 6.27E−06), *C1orf162* (p = 8.19E−05) and *NCAPD3* (p = 1.18E−04), which were highly significantly or highly upregulated, and then 0 mM group and 0.4 mM group including the genes *ALOX5AP* (p = 9.87E−12), *RNASET2* (p = 1.04E−12), *CLEC11A* (p = 1.07E−07), *C3AR1* (p = 7.99E−07) and *IRF9* (p = 2.06E−05), which were highly significantly or highly upregulated (Fig. 2c,d).

Clustering analysis of DEGs by WGCNA identified 10 co-expressed gene modules in MMNK-1 cholangiocytes and 9 co-expressed gene modules in THP-1 macrophages (Figs.1e and 2e). In the co-expressed gene modules for MMNK-1 cholangiocytes, we found that ME2, ME4, ME8 and ME9 modules showed monotonous increase or decrease in gene expression with level of 1,2-DCP (Fig. 1e). In the co-expressed gene modules for THP-1 macrophages, we found that ME2, ME3, ME8 and ME9 modules showed monotonous increase or decrease with level of 1,2-DCP (Fig. 2e).

Further analysis was conducted to evaluate the relevant processes and pathways involved in the transcriptomic profiles of co-cultures of MMNK-1 cholangiocytes and THP-1 macrophages exposed to 1,2-DCP for 24 h. For this purpose, overrepresentation analysis of gene ontology (GO) (biological process, cellular component and molecular function) and KEGG pathway terms, were employed. In MMNK-1 cells, significantly enriched (FDR < 0.05) term was detected only in the ME9 module (Fig. 1f and Table 1). “Base excision repair” of KEGG pathway term was highly enriched in the ME9 module, showing overrepresentation of *LIG1, PARP4*, and *POLD1* genes in the 1,2-DCP exposed group compared to the control group (Table 1).

**Table 1:** KEGG pathway overrepresentation analysis of ME9 module and list of involved genes in MMNK-1 cholangiocytes co-cultured with THP-1 macrophages.

Gene set	Description	Size	Expected	Observed	Ratio	p value	FDR
*hsa03410*	Base excision repair	33	8.84E−02	3	34.0	8.5E−05	0.028

Genes involved in base excision repair	Regulation	p-value	q-value	Mean ± SD of normalized values fold change			
				1,2-DCP concentration			
				0	0.1	0.4 mM	
DNA ligase 1 (*LIG1*)	Upregulated	5.3E−03	0.32	157 ± 13 1	175 ± 15 1.12	213.0 ± 3.4 1.36	
Poly (ADP-ribose) polymerase family member 4 (*PARP4*)	Upregulated	1.4E−03	0.19	199 ± 15 1	192 ± 30 0.96	271 ± 42 1.36	
DNA polymerase delta 1, catalytic subunit (*POLD1*)	Upregulated	2.3E−02	0.53	128 ± 19 1	127 ± 11 0.99	168 ± 19 1.31	

The table shows KEGG pathway overrepresentation analysis of ME9 module and involved genes from the transcriptomic profiles of MMNK-1 cholangiocytes co-cultured with THP-1 macrophages when exposed to 1,2-DCP at 0, 0.1 or 0.4 mM for 24 h. The analysis was peformed by WebGestalt with False Discovery Rate (FDR) cut-off value of 0.05. Data of normalized values for gene expression are mean ± SD, n = 3. Fold change represents the value relative to the average of the control group (0mM). All p-values for gene expression were adjusted using Benjamini-Hochberg method and expressed as q-values.

In THP-1 macrophages, significantly enriched (FDR < 0.05) terms were detected only in the ME2 module (Fig. 2f, Tables 2 and 3). The cell cycle-related GO process/KEGG pathway terms were highly enriched in the ME2 module (Tables 2 and 3). The genes annotated to the KEGG pathway included *BUBIB, CCNB2, CDC20, CDC45, CDK1, CDC7, MCM3, PLK1*, and *PTTG1*, which were all upregulated in the 1,2-DCP group, compared to the control group (Table 3). The genes annotated to various GO terms for biological processes of the ME2 module in THP-1 macrophages included those related to the mitosis (Supplementary Table S1), cell cycle (Supplementary Table S2), organelle fission (Supplementary Table S3), regulation of transferase activity (Supplementary Table S4), membrane invagination (Supplementary Table S5), chromosome organization (Supplementary Table S6) and NAD biosynthesis process (Supplementary Table S7). GO terms for cellular component (chromosome, microtubule cytoskeleton, inclusion body, cytoplasmic vesicle part and transferase complex) of ME2 module were significantly enriched (Supplementary Table S8).

**Table 2:** GO (biological process) overrepresentation analysis of ME2 module in THP-1 macrophages co-cultured with MMNK-1 cholangiocytes.

Gene set	Description	Size	Expected	Observed	Ratio	p-value	FDR
GO:1903047	Mitotic cell cycle process	788	8.70	33	3.79	3.2E−11	2.9E−07
GO:0007049	Cell cycle	1739	19.20	44	2.29	1.0E−07	1.3E−04
GO:0048285	Organelle fission	459	5.07	19	3.75	8.1E−07	6.1E−04
GO:0051338	Regulation of transferase activity	940	10.38	27	2.60	4.7E−06	2.7E−03
GO:0010324	Membrane invagination	60	0.66	6	9.06	5.1E−05	1.1E−02
GO:0051276	Chromosome organization	1143	12.62	27	2.14	1.4E−04	2.3E−02
GO:0009435	NAD biosynthetic process	53	0.59	5	8.54	2.9E−04	4.0E−02

The table shows GO (biological process) overrepresentation analysis of ME2 module from the transcriptomic profiles of THP-1 macrophages co-cultured with MMNK-1 cholangiocytes when exposed to 1,2-DCP at 0, 0.1 or 0.4 mM for 24 h. The analysis was performed by WebGestalt with False Discovery Rate (FDR) cut-off value of 0.05.

**Table 3:** KEGG pathway overrepresentation analysis of ME2 module and list of involved genes in THP-1 macrophages co-cultured with MMNK-1 cholangiocytes.

Gene set	Description	Size	Expected	Observed	Ratio	p-value	FDR
hsa04110	Cell cycle	124	1.63	9	5.53	3.3E−05	1.1E−02

Genes involved in cell cycle	Regulation	p-value	q-value	Mean ± SD of normalized values fold change			
				1,2-DCP concentration			
				0	0.1	0.4 mM	
BUB1 mitotic checkpoint serine/threonine kinase B (*BUB1B*)	Upregulated	6.9E−05	0.02	56 ± 10 1	89.1 ± 5.8 1.60	110 ± 30 1.98	
Cyclin B2 (*CCNB2*)	Upregulated	9.8E−04	0.08	36.1 ± 7.9 1	53.6 ± 4.9 1.49	70 ± 15 1.94	
Cell division cycle 20 (*CDC20*)	Upregulated	6.6E−04	0.21	164 ± 11 1	224 ± 41 1.37	233 ± 50 1.42	
Cell division cycle 45 (*CDC45*)	Upregulated	3.5E−02	0.42	64.1 ± 8.6 1	92.1 ± 5.6 1.44	76 ± 16 1.19	
Cell division cycle 7 (*CDC7*)	Upregulated	6.9E−03	0.21	15.8 ± 4.6 1	29.6 ± 5.0 1.87	30.9 ± 1.0 1.95	
Cyclin dependent kinase 1 (*CDK1*)	Upregulated	1.2E−06	7.69E−04	162 ± 14 1	240 ± 17 1.48	248 ± 15 1.53	
Minichromosome maintenance complex component 3(*MCM3*)	Upregulated	6.4E−03	0.21	166 ± 43 1	229 ± 24 1.38	234 ± 19 1.41	
Polo like kinase 1 (*PLK1*)	Upregulated	4.7E−02	0.46	40.5 ± 5.5 1	54.7 ± 4.6 1.35	60.0 ± 9.3 1.48	
Pituitary tumor-transforming 1 (*PTTG1*)	Upregulated	2.5E−03	0.14	162 ± 15 1	236 ± 42 1.46	212 ± 32 1.31	

The table shows KEGG pathway overrepresentation analysis of ME2 module and involved genes from the transcriptomic profiles of THP-1 macrophages co-cultured with MMNK-1 cholangiocytes when exposed to 1,2-DCP at 0, 0.1 or 0.4 mM for 24 h. The analysis was performed by WebGestalt with False Discovery Rate (FDR) cut-off value of 0.05. Data of normalized values for gene expression are mean ± SD, n = 3. All p-values for gene expression were adjusted using Benjamini–Hochberg method and expressed as q-values. Fold change represents the value relative to the average of the control group (0 mM).

### Genes with 1,2-DCP-induced dose-dependent changes in expression in MMNK-1 cholangiocytes and THP-1 macrophages

To identify those genes in MMNK-1 cholangiocytes and THP-1 macrophages whose expression levels were altered dose-dependently by exposure to 1,2-DCP, we further determined the effects of 1,2-DCP on the expression level using Pearson correlation coefficient. Tables 4 and 5 list the top and bottom five genes with the largest fold change at 0.4 mM relative to 0 mM 1,2-DCP. The genes whose expression levels were dose-dependently upregulated in MMNK-1 cholangiocytes were *ALDH2, PDE7B, LIG1, FN1*, and *GTF2E2* and those dose-dependently downregulated were *SNORD32A, HIST3H2BB, ENTPD5, SGO2* and *NABP2* (Table 4). On the other hand, the genes whose expression showed significant changes in response to 1,2-DCP in co-cultured THP-1 macrophages were *GUCY1B1, CD48, ULK4, KLRC1*, and *RGS13* while the dose-dependently downregulated genes were *SLC5A12, PWAR6, DAB1, AGO4*, and *ICAM5* (Table 5).

**Table 4:** Genes whose expression changed dose-dependently following exposure to 1,2-DCP in MMNK-1 cholangiocytes co-cultured with THP-1 macrophages.

Genes	p-value	q-value	Mean ± SD of normalized values fold change		
			1,2-DCP concentration		
			0	0.1	0.4 mM
**Upregulated**					
Aldehyde dehydrogenase 2 family (*ALDH2*)	4.2E−05	4.5E−02	7.4 ± 2.3 1	7.5 ± 2.4 1.01	18.2 ± 2.3 2.27
Phosphodiesterase 7B(*PDE7B*)	3.9E−05	4.4E−02	10.6 ± 1.7 1	15.7 ± 5.0 1.48	25.0 ± 1.7 2.36
DNA Ligase 1 (*LIG1*)	1.6E−05	2.8E−02	157 ± 13 1	175 ± 15 1.12	213.0 ± 3.4 1.36
Fibronectin 1 (*FN1*)	1.9E−05	2.9E−02	338.0 ± 5.3 1	375 ± 36 1.11	458 ± 24 1.35
General transcription factor IIE subunit 2 (*GTF2E2*)	5.2E−05	4.9E−02	63.4 ± 7.3 1	67.1 ± 4.0 1.06	85.5 ± 4.4 1.35
**Downregulated**					
Small nucleolar RNA, C/D box 32A (*SNORD32A*)	1.1E−05	2.2E−02	6.2 ± 1.7 1	4.16 ± 0.78 0.68	0.31 ± 0.53 0.05
Histone cluster 3 H2B family member b (*HIST3H2BB*)	5.6E−05	4.9E−02	4.7 ± 1.3 1	4.57 ± 0.75 0.98	0.69 ± 0.61 0.15
Ectonucleoside triphosphate diphosphohydrolase 5 (*ENTPD5*)	5.8E−05	4.9E−02	56.9 ± 4.2 1	55.8 ± 8.3 0.98	32.1 ± 4.0 0.57
Shugoshin 2 (*SGO2*)	8.6E−06	1.9E−02	442 ± 19 1	415 ± 27 0.94	339 ± 12 0.77
Nucleic acid binding protein 2 (*NABP2*)	3.6E−05	4.3E−02	486 ± 17 1	467.3 ± 5.6 0.96	434.0 ± 8.3 0.89

We tested the significance of Pearson correlation coefficient between the expression of each gene and 1,2-DCP level.

The table lists the top or bottom five genes with the largest fold change at 0.4 mM in MMNK-1 cholangiocytes co-cultured with THP-1 macrophages when exposed to 1,2-DCP at 0, 0.1 or 0.4 mM for 24 h. Data of normalized values for gene expression are mean ± SD, n = 3. All p-values for gene expression were adjusted using Benjamini–Hochberg method and expressed as q-values. Fold change represents the value relative to the average of the control group (0 mM).

**Table 5:** Genes whose expression changed dose-dependently following exposure of 1,2-DCP to THP-1 macrophages co-cultured with MMNK-1 cholangiocytes.

Genes	p-value	q-value	Mean ± SD of normalized values fold change		
			1,2-DCP concentration		
			0	0.1	0.4 mM
**Upregulated**					
Guanylate Cyclase 1 Soluble Subunit Beta 1 (*GUCY1B1*)	1.5E−04	4.6E−02	0.31 ± 0.54 1	2.8 ± 1.1 8.87	4.9 ± 1.0 15.62
CD48 molecule (*CD48*)	1.7E−05	2.1E−02	0.33 ± 0.58 1	0.38 ± 0.65 1.12	3.12 ± 0.29 9.33
unc-51 like kinase 4 (*ULK4*)	2.2E−05	2.3E−02	4.00 ± 0.88 1	8.3 ± 4.5 2.06	17.5 ± 2.0 4.37
killer cell lectin like receptor C1 (*KLRC1*)	5.5E−06	1.0E−02	1.6 ± 1.0 1	3.23 ± 0.14 1.98	5.50 ± 0.07 3.37
regulator of G-protein signaling 13 (*RGS13*)	1.6E−04	4.8E−02	5.6 ± 4.0 1	4.2 ± 2.6 0.75	17.6 ± 1.1 3.13
**Downregulated**					
solute carrier family 5 member 12 (*SLC5A12*)	9.7E−05	4.0E−02	6.4 ± 1.9 1	4.0 ± 1.4 0.62	0.37 ± 0.64 0.06
Prader Willi/Angelman region RNA 6 (*PWAR6*)	4.0E−05	2.8E−02	2.01 ± 0.14 1	1.98 ± 0.34 0.98	0.31 ± 0.54 0.15
DAB1, reelin adaptor protein (*DAB1*)	4.1E−05	2.7E−02	5.67 ± 0.32 1	5.2 ± 1.9 0.91	1.04 ± 0.10 0.18
Argonaute 4, RISC catalytic component (*AGO4*)	2.3E−05	2.2E−02	18.7 ± 2.1 1	17.4 ± 4.5 0.93	4.8 ± 1.7 0.26
Intercellular adhesion molecule 5 (*ICAM5*)	1.5E−04	4.6E−02	9.7 ± 1.6 1	8.6 ± 2.6 0.89	2.8 ± 1.4 0.29

We tested the significance of Pearson correlation coefficient between the expression of each gene and 1,2-DCP level.

The table lists the top or bottom five genes with the largest fold change at 0.4 mM in THP-1 macrophages co-cultured with MMNK-1 cholangiocytes when exposed to 1,2-DCP at 0, 0.1 or 0.4 mM for 24 h. Data of normalized values for gene expression are mean ± SD, n = 3. All p-values for gene expression were adjusted using Benjamini–Hochberg method and expressed as q- values. Fold change represents the value relative to the average of the control group (0 mM).

### Hypothesis-driven analysis shows that inflammatory responses are induced by exposure to 1,2-DCP in THP-1 macrophages but not in MMNK-1 cholangiocytes

Genes of the cytokines/chemokines, cytokine-related proteins, cytokine/chemokine receptors, whose expression was significantly different between levels of exposure to 1,2-DCP, in co-cultured THP-1 macrophages included cytokines (*TNFSF4, TNFAIP8L1, TNFAIP8L2-SCNM1* and receptor *TNFRSF10A*) and chemokines (*CXCL2, CCL2, CCL7* and receptors *CX3CR1, CCR6, CCRL2*), but ANOVA showed significant difference between levels of exposure only in *TNFAIP8L1, CCL2, CXCL2, CX3CR1* and *CCR6* (Table 6). None of interleukins or their receptors in THP-1 macrophages were significantly changed in expression. With regards to the cytokines, *TNFAIP8L1* was downregulated in the 1,2-DCP group compared to the control group. Among the chemokines, *CCL2* and receptors *CX3CR1* were upregulated in the 1,2-DCP exposed group, compared to the control group, whereas *CXCL2* and receptor *CCR6* were downregulated (Table 6). No genes of cytokines of TNF superfamily or interleukins, chemokines (*CCL, CXCL, CL* and *CX3CL*), cytokine/chemokine-related proteins and cytokine/chemokine receptors were differentially expressed between levels of exposure to 1,2-DCP in MMNK-1 cholangiocytes.

**Table 6:** ANOVA for expression levels of cytokines/chemokines-related genes or their receptors, which are selected by hypothesis, in THP-1 macrophages co-cultured with MMNK-1 cholangiocytes.

Genes involved in intercellular signal	Regulation	Module eigengene	p-value for ANOVA	Mean ± SD of normalized values fold change		
				1,2-DCP concentration		
				0	0.1	0.4 mM
**Cytokines**						
TNF Superfamily Member 4 (*TNFSF4*)	–	ME8	0.11	81 ± 16 1	52 ± 10 0.64	54 ± 19 0.67
Tumor necrosis factor alpha-induced protein 8-like protein 1 (*TNFAIP8L1*)	Downregulated	ME2	0.020	46.5 ± 8.4 1	27.3 ± 1.2 0.59 (0.013)	35.6 ± 5.6 0.77 (0.11)
Tumor necrosis factor, alpha-induced protein 8-like 2 and sodium channel modifier 1 (*TNFAIP8L2-SCNM1*)	–	ME3	0.15	150 ± 35 1	215 ± 47 1.43	200 ± 24 1.33
TNF Receptor Superfamily Member 10a (*TNFRSF10A*)	–	ME3	0.07	11.4 ± 4.1 1	14.7 ± 6.7 1.28	28 ± 10 2.42
**Chemokines**						
C–C motif chemokine ligand 2 (*CCL2*)	Upregulated	ME3	0.018	436 ± 83 1	575 ± 42 1.32 (0.057)	636 ± 52 1.46 (0.013)
C–C motif chemokine ligand 7(*CCL7*)	–	ME7	0.096	29.3 ± 6.5 1	33 ± 11 1.11	51 ± 13 1.73
C-X-C motif chemokine ligand 2 (*CXCL2*)	Downregulated	ME2	0.0042	27.8 ± 2.6 1	16.1 ± 4.4 0.58 (0.018)	10.6 ± 4.4 0.38 (0.0028)
C-X3-C motif chemokine receptor 1(*CX3CR1*)	Upregulated	ME2	0.0016	13.5 ± 5.5 1	30.6 ± 6.2 2.26 (0.024)	32.3 ± 6.4 2.39 (0.016)
C–C motif chemokine receptor like 2(*CCRL2*)	–	ME9	0.053	35 ± 13 1	50 ± 12 1.46	62.8 ± 7.1 1.82
C–C motif chemokine receptor 6(*CCR6*)	Downregulated	ME2	0.0083	30.7 ± 1.0 1	17.9 ± 4.5 0.58 (0.0074)	19.8 ± 3.9 0.64 (0.015)

Normalized values of expression level were compared between three groups of different 1,2-DCP concentration by one-way analysis of variance (ANOVA), being followed by *post hoc* Dunnett’s multiple comparison with control (0 mM 1,2-DCP group). Data represents expression levels of cytokines/chemokines-related genes or their receptors from transcriptomic profiles of THP-1 macrophages co-cultured with MMNK-1 cholangiocytes when exposed to 1,2-DCP at 0, 0.1 or 0.4 mM for 24 h. Data of normalized values for gene expression are mean ± SD, n = 3. Fold change represents the value relative to the average of the control group (0 mM).

### ANOVA for expression levels of genes categorized for base excision repair (BER), homologous recombination (HR) and non-homologous end joining (NHEJ) pathway 

Among differentially expressed genes categorized for base excision repair pathway, *LIG1, PARP4, POLD1* and *OGG1* showed significant difference in expression level between levels of 1,2-DCP exposure and upregulated by exposure to 1,2-DCP (Supplementary Table S9). Regarding genes categorized for homologous recombination (HR) pathway, *NBN* and *RPA1* were upregulated by exposure to 1,2-DCP, but no genes categorized for non-homologous end joining (NHEJ) pathway showed significant change in expression level between levels of exposure to 1,2-DCP.

## Discussion

In this study, we investigated the transcriptomic profiles of co-cultures of MMNK-1 cholangiocytes and THP-1 macrophages after 24-h exposure to 1,2-DCP. We used the co-culture model of cholangiocytes and macrophages to mimic an inflammatory environment and exposed these groups of cells to 1,2-DCP to determine the transcriptional activities that occur under such an environment thereby identifying changes or processes occurring within cholangiocytes and macrophages leading to DNA damage, which is thought to play a pivotal role in carcinogenesis.

Immunohistochemical analysis of specimens of occupational cholangiocarcinoma showed high infiltration of inflammatory cells, even at sites of the bile duct in noncancerous hepatic tissues^3^. Importantly, Trush and Kensler reported increased toxicity of chemicals in the presence of inflammatory cells^33^. Furthermore, our group recently demonstrated the important role of macrophages in 1,2-DCP-induced cytotoxicity, reactive oxygen species production and DNA damage in cholangiocytes exposed to 1,2-DCP, which occurred only in the presence of macrophages^11,14^. As such, we sought to identify the transcriptional activities associated with the increased cytotoxic and genotoxic effects of 1,2-DCP, on co-cultured MMNK-1 cholangiocytes/THP-1 macrophages, to enhance our understanding of the molecular mechanisms involved in 1,2-DCP-induced cholangiocarcinoma.

In this study, we used 1,2-DCP concentration range comparable to the 1,2-DCP exposure levels experienced by the workers of the printing companies in Japan, who were diagnosed with occupational cholangiocarcinoma^2^, as described in the “Materials and methods” section.

KEGG pathway enrichment analysis showed base excision repair term was enriched in line with increase in 1,2-DCP level in the co-cultured MMNK-1 cholangiocytes (Table 1, Fig. 1e). Furthermore, it also showed upregulation of all the genes annotated to base excision repair in the 1,2-DCP group compared to the control group (Table 1), consistent with our previous reports that DNA damage occurred in co-cultured MMNK-1 cholangiocytes following exposure to 1,2-DCP^11,13,14^.

Carcinogenesis occurs in three stages, namely: initiation, promotion, and progression. DNA damage has been established as the event that initiates carcinogenesis^34,35^. Faults in the DNA repair systems could also burden the cells with potential disadvantageous mutations^36^. More strand breaks could occur during the repair process, which could further enhance genomic instability or cell death^37^. It is therefore inferred that increased DNA damage can both enhance and compromise the survival of initiated cells when some damaged DNA escapes DNA repair or is left not fully repaired^34^.

In addition to DNA damage, initiation of cancers is enhanced in the presence of increased DNA damaging agent^34,38^. The upregulation of DNA repair genes suggests increase in DNA damage as 1,2-DCP concentration is increased, which could enhance mutation in the cells thereby increasing the resultant neoplasia. Moreover, the DNA damage in cholangiocytes co-cultured with THP-1 macrophages has been shown to be 1,2-DCP dose-dependent^14^. Immunohistological analysis of specimens obtained from the 1,2-DCP cholangiocarcinoma cases showed overexpression of γH2AX, a marker of DNA double-strand break, in the foci of BilIN, IPNB, invasive carcinoma, and non-neoplastic biliary epithelial cells, compared to specimen from control of common cholangiocarcinoma^7^.

Overexpression or mutation of the base excision repair genes (*LIG1, PARP4* and *POLD1*), of which expression was upregulated by 1,2-DCP exposure (Table 1), has been linked to genomic instability, poor prognosis, and progression of cancer^39–42^.

The transcriptomic profiling of THP-1 macrophages co-cultured with MMNK-1 cholangiocytes exposed to 1,2-DCP indicated that enrichment of cell cycle related processes was associated with 1,2-DCP exposure (Tables 2 and 3). All the expression of the genes (*BUBIB, CCNB2, CDC20, CDC45, CDK1, CDC7, MCM3, PLK1*, and *PTTG1*) associated with the enriched terms were upregulated in the 1,2-DCP group, compared to the control group (Table 3). Because most of these genes are particularly engaged in ensuring the progression of the cell cycle from G1 to S and from G2 to M, and ensuring the proliferation of the cells^43,44^, exposure of co-cultures of THP-1 macrophages and MMNK-1 cholangiocytes to 1,2-DCP might induce the proliferation of the THP-1 macrophages. Because macrophages have the major role in the regulation of inflammatory responses and a subset of macrophages could locally proliferate^45^, accumulation of macrophages at the site of injury following exposure to 1,2-DCP possibly affects inflammatory responses, carcinogenesis, and tumor microenvironment.

Further analysis showed significant and dose-dependent changes in the expression of genes in the overall transcriptomic profiles of 1,2-DCP-exposed MMNK-1 cholangiocytes/THP-1 macrophages co-cultures (Tables 4 and 5). *LIG1* (which was also found to be a component of the base excision repair pathway in KEGG analysis of MMNK-1 cholangiocytes) and *FN1*, which are implicated in diseases and cancer, were significantly correlated genes with the increase in 1,2-DCP level in the co-cultured MMNK-1 cells. DNA ligase 1 (*LIG1*) gene encodes a member of the ATP-dependent DNA ligase protein family, which plays a role in DNA replication, recombination, and repair pathways where it seals nicks in double stranded DNA^46^. Furthermore, previous studies demonstrated the engagement of *LIG1* in various repair pathways, such as short-patch^47^ or long-patch^48^ base-excision repair, nucleotide excision repair^49^, mismatch repair^50^ and non-homologous end-joining^51,52^. In pathological conditions, upregulation of *LIG1* expression has been demonstrated in many human cancers^42^ and mutations in *LIG1* gene are associated with retarded joining of Okazaki fragments during DNA replication, hypersensitivity to a variety of DNA-damaging agents and aberrant DNA repair in human fibroblast strain (46BR) cells^53–55^. Fibronectin1(*FN1*) encodes a dimeric glycoprotein known to function in cell adhesion, cytoskeletal organization, migration, proliferation, and differentiation^56,57^. High *FN1* levels have been associated with increased invasion and metastatic capability in lung and hepatic cancers^56,58^. It has also been reported to be a causative factor in the development of various pathological conditions, such as liver cirrhosis^59^. FN1 is also reported to stimulate the expression of various inflammatory factors in the tumor microenvironment, thereby highlighting the regulatory influence of this glycoprotein in major inflammatory cells^60,61^.

The results of hypothesis-driven gene expression analysis suggest the expression changes of TNF-α-induced proteins *TNFAIP8L1*, as well as chemokines *CCL2* and receptors *CX3CR1, CCR6* occurred in THP-1 macrophages (Table 6) by exposure to 1,2-DCP, but not in MMNK-1 cholangiocytes. Our previous studies showed monocultured cholangiocytes exposed to 1,2-DCP showed no significant change in expression of γ-H2AX, suggesting the involvement of macrophages in the induction of increased DNA damage^14^. While the TNF-α related proteins remain strong candidates for extracellular signaling involved in DNA damage in cholangiocytes, further studies are needed to clarify their exact roles and cross talk between cholangiocytes and macrophages in 1,2-DCP-induced DNA damage in cholangiocytes.

Generally, activation of macrophages leads to the release of cytokines and chemokines, which contributes to crosstalk between the macrophages and their environment^62^. Since the primary function of cytokines is the regulation of immune and inflammatory responses of the host to the invading foreign substances or tissue injury, they play a vital role in ensuring the overall health of the host^63^. Repeated exposure to toxicants or xenobiotics induces persistent production of cytokines and chemokines by macrophages, resulting in enhancement of inflammation, trying to control cellular stress and minimize cellular damage^64^. However, this could induce dysregulated cytokine and chemokine production, which could subsequently result in various pathological states and cancer^63,65^. We have demonstrated recently that 24-h exposure of THP-1 macrophages to 1,2-DCP results in upregulation of TNF-α, IL-1β and IL-6 protein expression^11,14^. In this study, the mRNA expression levels of *TNF-*α*, IL-1β*, and *IL-6* were not significantly changed in the cell co-cultures. Previous studies have shown differences between expression changes in monocultured cells and when in co-culture with other cells^66^ and differences between mRNA expression and protein expression^67^. However, the expression levels of certain tumor necrosis factor superfamilies of ligands (TNFSF) and receptors (TNFRSF) such as *TNFSF4*, and *TNFRSF10A*, and tumor necrosis factor-α-induced protein 8-like family (TIPE) such as *TNFAIP8L1, TNFAIP8L2-SCNM1* were changed. TNFSF and TNFRSF are known to be expressed by or target immune cells such as macrophages and some non-immune cells through co-stimulatory and inhibitory pathways to induce the expression of wide range of actions including cellular differentiation, survival, and production of inflammatory cytokines and chemokines^68–70^. Active TNFSF ligand–receptor signaling pathways are associated with inflammatory disease and cancer^68,71^. More specifically, low expression of *TNFSF4* mRNA was associated with worse prognosis in melanoma patients^71^. TIPE family has been described as regulators of immunity and tumorigenesis^72^. An expression analysis in humans showed that the TIPE family is dysregulated in cancer and inflammation and plays critical role in tumorigenesis and inflammatory responses^73^. *TNFAIP8L1* has been demonstrated as an inducer of cell death^74^. Previous study showed the down regulation of *TNFAIP8L1* in hepatocellular carcinoma (HCC) tissues which correlated with tumor pathogenic grade and patient survival^74^. In this study, interestingly 1,2-DCP exposed co-cultured macrophages showed a downregulation of *TNFAIP8L1* in the 1,2-DCP exposed cells compared to the control group.

Among genes categorized for base excision repair, only *LIG1, PARP4* and *POLD1* are differentially expressed in ME 9 but it should be noted that *OGG1*, which is categorized for base excision repair pathway, in ME7 is also upregulated by exposure to 1,2-DCP (Supplementary Table S9). Upregulation of base excision repair genes in cholangiocytes co-cultured with macrophages doesn’t indicate that 1,2-DCP-induced DNA damage is exclusively limited to DNA base damage. Other than base excision repair pathway, *LIG1* and *POLD1* are also related to replication and other DNA repair pathways and *PARP4* is also related to apoptosis or transportation as a vault protein. Our previous studies showed that exposure to 1,2-DCP increased DNA damage as assessed by alkaline comet assay, which detects both single strand breaks (SSBs) and double strand breaks (DSBs), and γH2AX expression, which detects DSBs, in cholangiocytes co-cultured with macrophages^11,14^.

KEGG pathway enrichment analysis did not detect significant involvement of homologous recombination repair (HRR) or non-homologous end joining (NHEJ) pathway. When looking at each gene listed in any module eigengene, *NBN* and *RPA1* in ME8 and *POLD1* in ME9 categorized for HRR were upregulated by exposure to 1,2-DCP, but no genes categorized for NHEJ pathway in any module eigengene showed significant 1,2-DCP exposure-related change in expression level (Supplementary Table S9). Collectively, the study does not exclude possible involvement of HRR pathway with repair of DNA lesions.

On the other hand, the result did not detect 1,2-DCP-induced upregulation of *AICDA* expression. This might be due to the difference in the length of exposure between the present study and the previous study, as upregulation of *AICDA* was optimal after 9-h exposure to 1,2-DCP but fell down greatly after 12-h exposure to 1,2-DCP^13^, thus the present study does not disprove possible involvement of *AICDA* with 1,2-DCP-induced DNA damage in MMNK-1 cholangiocytes co-cultured with THP-1 macrophages. Interestingly a recent study shows that base excision repair is required for the processing of AID-induced lesions into DNA double strand breaks^75^. Further studies are needed to clarify the role of AID in 1,2-DCP-induced DNA damage in cholangiocytes.

The mechanism of how 1,2-DCP induces DNA damage has not been revealed. Our previous studies showed increase in reactive oxygen species (ROS)^14^, tail DNA% and tail moment in comet assay, or AID expression^13^ in MMNK-1 cholangiocytes by co-culture with THP-1 macrophages, suggesting involvement of ROS or AID in DNA damage in cholangiocytes. Exposure to 1,2-DCP increased ROS level dose-dependently in MMNK-1 cholangiocytes co-cultured with THP-1 macrophages, but not in monocultured MMNK-1 cholangiocytes or THP-1 macrophages, suggesting ROS is produced by intrinsic mechanism in MMNK-1 cholangiocytes although it is activated by exposure to 1,2-DCP in the presence of macrophages^14^. Elevated ROS levels cause damage to DNA including abasic sites, single strand DNA breaks (SSBs), sugar moiety modifications, deaminated and adducted bases^76–78^. Oxidative base lesions such as highly mutagenic guanine derivative 7,8-dihydro-8-oxoguanine (8-oxoG) and the corresponding ring fragmented purine formamidopyrimidine derivative (FapyG) or abasic sites are predominantly repaired by base excision repair (BER) and to a lesser extent nucleotide excision repair^47,79,80^. Oxidative DNA lesions can lead to DNA double-strand break (DSB) formation which is originated from single strand break (SSB) during repair, excision of base, topoisomerase cleavage, DNA replication or transcription^81–85^. Upregulation of BER genes in the present study may be a response to ROS-induced DNA damage, although exact mechanism on how DSBs are generated is not clarified. Given the fact that exposure to 1,2-DCP increases the number of cholangiocytes with γH2AX-positive foci or the number of γH2AX-positive foci per nucleus of cholangiocytes, suggesting occurrence of DSB in cholangiocytes, in the presence of macrophages^11,14^, it is likely that various pathways of DNA damage repair may be involved. Studies using cells with pathway-specific gene-knockouts are needed to fully understand how efficient but occasionally erroneous DNA damage repair occurs in cholangiocytes exposed to 1,2-DCP in the presence of macrophages.

## Conclusions

The transcriptomic profiles of MMNK-1 cholangiocytes showed that the upregulation of base excision repair genes, and that such upregulation was 1,2-DCP-concentration dependent, indicating increased DNA damage in the cholangiocytes. The transcriptomic profiles of THP-1 macrophages, however; showed upregulation of cell cycle-related genes, indicating enhanced proliferation of macrophages. Upregulation of the base excision repair genes might be involved in the previously observed DNA damage in MMNK-1 cholangiocytes co-cultured with THP-1 macrophages, although involvement of other mechanisms such as DNA replication, cell death or other types of DNA repair was not disproved. Cross talk interactions between cholangiocytes and macrophages explaining the observed increase in DNA damage in the cholangiocytes should be explored further.

## Supplementary Information

Supplementary Tables.

## Data Availability

Raw data, processed data and metadata of transcriptome analysis have been deposited in NCBI Gene Expression Omnibus (GEO; http://www.ncbi.nlm.nih.gov/geo; GSE 198858).
