# Supplementary material for: Transcriptome analysis of human cholangiocytes exposed to carcinogenic 1,2-dichloropropane in the presence of macrophages in vitro
Source: Sci Rep. 2022 Jul 2;12:11222. doi: 10.1038/s41598-022-15295-3 (PMC9250500; doi:10.1038/s41598-022-15295-3)
Supplement: Supplementary file 1 — Supplementary Tables. [file 41598_2022_15295_MOESM1_ESM.docx]

# Supplementary Information

Supplementary Tables S1 - S7, shows the listed genes for the GO Terms-Biological processes of ME2 gene module, co-cultured THP-1 macrophages.

Supplementary Table S8, shows the GO Terms-Cellular components of ME2 module, co-cultured THP-1 macrophages.

Supplementary Table S9, shows ANOVA for expression level of genes categorized for base excision repair (BER), homologous recombination (HR) and non-homologous end joining pathway.

Supplementary Table S1. Mitotic cell cycle-related genes- GO term Biological Process of ME2 gene module, co-cultured THP-1 macrophages

| **Genes** | **Regulation** | **p value** | **q value** |
| --- | --- | --- | --- |
| aurora kinase B (*AURKB*) | Upregulated | 1.8E-02 | 0.31 |
| bone morphogenetic protein 7 (*BMP7*) | Downregulated | 2.1E-02 | 0.34 |
| BUB1 mitotic checkpoint serine/threonine kinase B (*BUB1B*) | Upregulated | 6.9E-05 | 0.02 |
| cyclin B2 (*CCNB2*) | Upregulated | 9.8E-04 | 0.08 |
| cell division cycle 20 (*CDC20*) | Upregulated | 6.6E-03 | 0.21 |
| cell division cycle 45 (*CDC45*) | Upregulated | 3.5E-02 | 0.42 |
| cell division cycle 7 (*CDC7*) | Upregulated | 6.9E-03 | 0.21 |
| cyclin dependent kinase 1 (*CDK1*) | Upregulated | 1.2E-06 | 7.69E-04 |
| CDC28 protein kinase regulatory subunit 1B (*CKS1B*) | Upregulated | 6.8E-03 | 0.21 |
| CCR4-NOT transcription complex subunit 6 like (*CNOT6L*) | Upregulated | 2.6E-02 | 0.37 |
| DLG associated protein 5 (*DLGAP5*) | Upregulated | 6.9E-03 | 0.21 |
| DNA replication and sister chromatid cohesion 1(*DSCC1*) | Upregulated | 2.4E-02 | 0.35 |
| DSN1 homolog, MIS12 kinetochore complex component (*DSN1*) | Upregulated | 7.0E-03 | 0.21 |
| H2A histone family member Y (*H2AFY*) | Upregulated | 2.2E-02 | 0.34 |
| Huntingtin (*HTT*) | Upregulated | 2.8E-02 | 0.38 |
| kinesin family member C1 (*KIFC1*) | Upregulated | 1.7E-02 | 0.31 |
| minichromosome maintenance complex component 3 (*MCM3*) | Upregulated | 6.4E-03 | 0.21 |
| migration and invasion inhibitory protein (*MIIP*) | Upregulated | 1.8E-03 | 0.12 |
| non-SMC condensin II complex subunit D3(*NCAPD3*) | Upregulated | 1.9E-04 | 0.03 |
| NDC80 kinetochore complex component NUF2 (*NUF2*) | Upregulated | 3.4E-03 | 0.16 |
| nucleolar and spindle associated protein 1(*NUSAP1*) | Upregulated | 2.0E-02 | 0.33 |
| polo like kinase 1 (*PLK1*) | Upregulated | 4.7E-02 | 0.46 |
| protein phosphatase 3 catalytic subunit alpha (*PPP3CA*) | Upregulated | 1.6E-03 | 0.11 |
| protein regulator of cytokinesis 1 (*PRC1*) | Upregulated | 1.6E-02 | 0.30 |
| protein kinase cAMP-dependent type II regulatory subunit beta (*PRKAR2B*) | Downregulated | 4.2E-02 | 0.45 |
| protein kinase C alpha (*PRKCA*) | Downregulated | 4.8E-02 | 0.47 |
| pituitary tumor-transforming 1 (*PTTG1*) | Upregulated | 2.5E-03 | 0.14 |
| schlafen family member 11 (*SLFN11*) | Upregulated | 2.4E-02 | 0.36 |
| sorting nexin 18 (*SNX18*) | Downregulated | 2.9E-02 | 0.39 |
| stathmin 1 (*STMN1*) | Upregulated | 2.3E-03 | 0.13 |
| SYF2 pre-mRNA splicing factor (*SYF2*) | Downregulated | 3.3E-03 | 0.16 |
| ubiquitin conjugating enzyme E2 C (*UBE2C*) | Upregulated | 5.1E-05 | 0.01 |
| ZW10 interacting kinetochore protein (*ZWINT*) | Upregulated | 1.7E-04 | 0.03 |

p values were adjusted using Benjamini-Hochberg method and expressed as q value.

Supplementary Table S2. Cell cycle-related genes- GO term Biological Process of ME2 gene module, co-cultured THP-1 macrophages

| **Genes** | **Regulation** | **p value** | **q value** |
| --- | --- | --- | --- |
| amyloid beta precursor protein binding family B member 2 (*APBB2*) | Downregulated | 2.5E-02 | 0.36 |
| aurora kinase B (*AURKB*) | Upregulated | 1.8E-02 | 0.31 |
| bone morphogenetic protein 7 (*BMP7*) | Downregulated | 2.1E-02 | 0.34 |
| BUB1 mitotic checkpoint serine/threonine kinase B (*BUB1B*) | Upregulated | 6.9E-05 | 0.02 |
| cyclin B2 (*CCNB2*) | Upregulated | 9.8E-04 | 0.08 |
| cell division cycle 20 (*CDC20*) | Upregulated | 6.6E-03 | 0.21 |
| cell division cycle 45 (*CDC45*) | Upregulated | 3.5E-02 | 0.42 |
| cell division cycle 7 (*CDC7*) | Upregulated | 6.9E-03 | 0.21 |
| cyclin dependent kinase 1 (*CDK1*) | Upregulated | 1.2E-06 | 7.7E-04 |
| cyclin dependent kinase 18 (*CDK18*) | Upregulated | 7.9E-03 | 0.23 |
| CDC28 protein kinase regulatory subunit 1B (*CKS1B*) | Upregulated | 6.8E-03 | 0.21 |
| CCR4-NOT transcription complex subunit 6 like (*CNOT6L*) | Upregulated | 2.6E-02 | 0.37 |
| DLG associated protein 5 (*DLGAP5*) | Upregulated | 6.9E-03 | 0.21 |
| DNA replication and sister chromatid cohesion 1(*DSCC1*) | Upregulated | 2.4E-02 | 0.35 |
| DSN1 homolog, MIS12 kinetochore complex component (*DSN1*) | Upregulated | 7.0E-03 | 0.21 |
| FA complementation group I (*FANCI*) | Upregulated | 1.1E-02 | 0.25 |
| H2A histone family member Y (*H2AFY*) | Upregulated | 2.2E-02 | 0.34 |
| Huntingtin (*HTT*) | Upregulated | 2.8E-02 | 0.38 |
| kinesin family member C1 (*KIFC1*) | Upregulated | 1.7E-02 | 0.31 |
| minichromosome maintenance complex component 3 (*MCM3*) | Upregulated | 6.4E-03 | 0.21 |
| migration and invasion inhibitory protein (*MIIP*) | Upregulated | 1.8E-03 | 0.12 |
| non-SMC condensin II complex subunit D3(*NCAPD3*) | Upregulated | 1.9E-04 | 0.03 |
| NDC80 kinetochore complex component NUF2 (*NUF2*) | Upregulated | 3.4E-03 | 0.16 |
| nucleolar and spindle associated protein 1(*NUSAP1*) | Upregulated | 2.0E-02 | 0.33 |
| polo like kinase 1(PLK1) | Upregulated | 4.7E-02 | 0.46 |
| DNA polymerase delta 1, catalytic subunit (*POLD1*) | Upregulated | 1.5E-02 | 0.29 |
| DNA polymerase delta 3, accessory subunit (*POLD3*) | Upregulated | 2.6E-02 | 0.37 |
| protein phosphatase 3 catalytic subunit alpha (*PPP3CA*) | Upregulated | 1.6E-03 | 0.11 |
| protein kinase cAMP-dependent type II regulatory subunit beta (*PRKAR2B*) | Downregulated | 4.2E-02 | 0.45 |
| protein kinase C alpha (*PRKCA*) | Downregulated | 4.8E-02 | 0.47 |
| pituitary tumor-transforming 1 (*PTTG1*) | Upregulated | 2.5E-03 | 0.14 |
| RAS like proto-oncogene A (*RALA*) | Downregulated | 4.3E-02 | 0.45 |
| Ras related GTP binding B (*RRAGB*) | Downregulated | 3.8E-02 | 0.43 |
| Ras related GTP binding D (*RRAGD*) | Downregulated | 4.4E-02 | 0.45 |
| spindle and kinetochore associated complex subunit 3(*SKA3*) | Upregulated | 4.9E-03 | 0.19 |
| schlafen family member 11 (*SLFN11*) | Upregulated | 2.4E-02 | 0.36 |
| sorting nexin 18 (*SNX18*) | Downregulated | 2.9E-02 | 0.39 |
| stathmin 1 (*STMN1*) | Upregulated | 2.3E-03 | 0.13 |
| SYF2 pre-mRNA splicing factor (*SYF2*) | Downregulated | 3.3E-03 | 0.16 |
| thrombospondin 1(*THBS1*) | Upregulated | 1.2E-03 | 0.10 |
| ubiquitin conjugating enzyme E2 C (*UBE2C*) | Upregulated | 5.1E-05 | 0.01 |
| WD repeat and HMG-box DNA binding protein 1 (*WDHD1*) | Upregulated | 4.2E-02 | 0.45 |
| ZW10 interacting kinetochore protein (*ZWINT*) | Upregulated | 1.7E-04 | 0.03 |

p values were adjusted using Benjamini-Hochberg method and expressed as q value.

Supplementary Table S3. Organelle fission-related genes- GO term Biological Process of ME2 gene module, co-cultured THP-1 macrophages

| **Genes** | **Regulation** | **p value** | **q value** |
| --- | --- | --- | --- |
| acyl-CoA thioesterase 8 (*ACOT8*) | Upregulated | 1.9E-02 | 0.32 |
| aurora kinase B (*AURKB*) | Upregulated | 1.8E-02 | 0.31 |
| bone morphogenetic protein 7 (*BMP7*) | Downregulated | 2.1E-02 | 0.34 |
| BUB1 mitotic checkpoint serine/threonine kinase B (*BUB1B*) | Upregulated | 6.9E-05 | 0.02 |
| cyclin B2 (*CCNB2*) | Upregulated | 9.8E-04 | 0.08 |
| cell division cycle 20 (*CDC20*) | Upregulated | 6.6E-03 | 0.21 |
| DLG associated protein 5 (*DLGAP5*) | Upregulated | 6.9E-03 | 0.21 |
| DNA replication and sister chromatid cohesion 1 (*DSCC1*) | Upregulated | 2.4E-02 | 0.35 |
| DSN1 homolog, MIS12 kinetochore complex component (*DSN1*) | Upregulated | 7.0E-03 | 0.21 |
| H2A histone family member Y (*H2AFY*) | Upregulated | 2.2E-02 | 0.34 |
| kinesin family member C1 (*KIFC1*) | Upregulated | 1.7E-02 | 0.31 |
| non-SMC condensin II complex subunit D31(*NCAPD3*) | Upregulated | 1.9E-04 | 0.03 |
| NDC80 kinetochore complex component NUF2 (*NUF2*) | Upregulated | 3.4E-03 | 0.16 |
| nucleolar and spindle associated protein 1 (*NUSAP1*) | Upregulated | 2.0E-02 | 0.33 |
| polo like kinase 1 (*PLK1*) | Upregulated | 4.7E-02 | 0.46 |
| protein regulator of cytokinesis 1 (*PRC1*) | Upregulated | 1.6E-02 | 0.30 |
| pituitary tumor-transforming 1 (*PTTG1*) | Upregulated | 2.5E-03 | 0.14 |
| ubiquitin conjugating enzyme E2 C (*UBE2C*) | Upregulated | 5.1E-05 | 0.01 |
| ZW10 interacting kinetochore protein (*ZWINT*) | Upregulated | 1.7E-04 | 0.03 |

p values were adjusted using Benjamini-Hochberg method and expressed as q value.

Supplementary Table S4. Regulation of transferase activity-related genes- GO term Biological Process of ME2 gene module, co-cultured THP-1 macrophages

| **Genes** | **Regulation** | **p value** | **q value** |
| --- | --- | --- | --- |
| aurora kinase B (*AURKB*) | Upregulated | 1.8E-02 | 0.31 |
| bone morphogenetic protein 7 (*BMP7*) | Downregulated | 2.1E-02 | 0.34 |
| cyclin B2 (*CCNB2*) | Upregulated | 9.8E-04 | 0.08 |
| CD74 molecule (*CD74*) | Upregulated | 7.6E-05 | 0.02 |
| cell division cycle 20 (*CDC20*) | Upregulated | 6.6E-03 | 0.21 |
| cyclin dependent kinase 1 (*CDK1*) | Upregulated | 1.2E-06 | 7.69E-04 |
| CDC28 protein kinase regulatory subunit 1B (*CKS1B*) | Upregulated | 6.8E-03 | 0.21 |
| DNA replication and sister chromatid cohesion 1 (*DSCC1*) | Upregulated | 2.4E-02 | 0.35 |
| dual specificity phosphatase 12 (*DUSP12*) | Upregulated | 5.0E-03 | 0.19 |
| fatty acid binding protein 4 (*FABP4*) | Upregulated | 3.4E-02 | 0.42 |
| growth differentiation factor 15 (*GDF15*) | Upregulated | 1.1E-03 | 0.09 |
| H2A histone family member Y (*H2AFY*) | Upregulated | 2.2E-02 | 0.34 |
| huntingtin (*HTT*) | Upregulated | 2.8E-02 | 0.38 |
| insulin receptor substrate 2 (*IRS2*) | Downregulated | 8.5E-03 | 0.24 |
| LIM domain only 4 (*LMO4*) | Downregulated | 1.9E-02 | 0.32 |
| lysophosphatidic acid receptor 1 (*LPAR1*) | Upregulated | 2.6E-02 | 0.37 |
| p21 (RAC1) activated kinase 1 (*PAK1*) | Upregulated | 1.2E-02 | 0.27 |
| poly(ADP-ribose) polymerase family member 16 (*PARP16*) | Downregulated | 5.0E-02 | 0.47 |
| polo like kinase 1 (*PLK1*) | Upregulated | 4.7E-02 | 0.46 |
| Protein kinase cAMP-dependent type II regulatory subunit beta (*PRKAR2B*) | Downregulated | 4.2E-02 | 0.45 |

p values were adjusted using Benjamini-Hochberg method and expressed as q value.

Supplementary Table S5. Membrane invagination-related genes- GO term Biological Process of ME2 gene module, co-cultured THP-1 macrophages.

| **Genes** | **Regulation** | **p value** | **q value** |
| --- | --- | --- | --- |
| aurora kinase B (*AURKB*) | Upregulated | 1.8E-02 | 0.31 |
| Fc fragment of IgG receptor Ia (*FCGR1A*) | Upregulated | 1.6E-05 | 6.1E-03 |
| sorting nexin 18 (*SNX18*) | Downregulated | 2.9E-02 | 0.39 |
| synaptotagmin 11 (*SYT11*) | Downregulated | 1.3E-02 | 0.28 |
| thrombospondin 1 (*THBS1*) | Upregulated | 1.2E-03 | 9.6E-02 |
| triggering receptor expressed on myeloid cells 2 (*TREM2*) | Upregulated | 4.9E-05 | 0.01 |

p values were adjusted using Benjamini-Hochberg method and expressed as q value.

Supplementary Table S6. Chromosome organization-related genes- GO term Biological Process of ME2 gene module, co-cultured THP-1 macrophages.

| **Genes** | **Regulation** | **p value** | **q value** |
| --- | --- | --- | --- |
| actin like 6A (*ACTL6A*) | Upregulated | 2.3E-03 | 0.13 |
| ATPase family, AAA domain containing 2 (*ATAD2*) | Upregulated | 1.9E-02 | 0.33 |
| aurora kinase B (*AURKB*) | Upregulated | 1.8E-02 | 0.31 |
| BUB1 mitotic checkpoint serine/threonine kinase B (*BUB1B*) | Upregulated | 6.9E-05 | 0.02 |
| cell division cycle 20 (*CDC20*) | Upregulated | 6.6E-03 | 0.21 |
| cell division cycle 45 (*CDC45*) | Upregulated | 3.5E-02 | 0.42 |
| cyclin dependent kinase 1 (*CDK1*) | Upregulated | 1.2E-06 | 7.7E-04 |
| centromere protein H (*CENPH*) | Upregulated | 6.4E-03 | 0.21 |
| centromere protein K (CENPK) | Upregulated | 1.1E-05 | 4.8E-03 |
| DLG associated protein 5 (*DLGAP5*) | Upregulated | 6.9E-03 | 0.21 |
| DNA replication and sister chromatid cohesion 1 (*DSCC1*) | Upregulated | 2.4E-02 | 0.35 |
| DSN1 homolog, MIS12 kinetochore complex component (*DSN1*) | Upregulated | 7.0E-03 | 0.21 |
| GINS complex subunit 2 (*GINS2*) | Upregulated | 2.8E-04 | 0.04 |
| H2A histone family member Y (*H2AFY*) | Upregulated | 2.2E-02 | 0.34 |
| high mobility group box 2 (*HMGB2*) | Upregulated | 1.2E-02 | 0.26 |
| kinesin family member C1 (*KIFC1*) | Upregulated | 1.7E-02 | 0.31 |
| non-SMC condensin II complex subunit D3 (*NCAPD3*) | Upregulated | 1.9E-04 | 0.03 |
| NDC80 kinetochore complex component NUF2 (*NUF2*) | Upregulated | 3.4E-03 | 0.16 |
| nucleolar and spindle associated protein 1 (*NUSAP1*) | Upregulated | 2.0E-02 | 0.33 |
| polo like kinase 1 (*PLK1*) | Upregulated | 4.7E-02 | 0.46 |
| DNA polymerase delta 1, catalytic subunit (*POLD1*) | Upregulated | 1.5E-02 | 0.29 |
| DNA polymerase delta 3, accessory subunit (*POLD3*) | Upregulated | 2.6E-02 | 0.37 |
| protein regulator of cytokinesis 1(*PRC1*) | Upregulated | 1.6E-02 | 0.30 |
| protein kinase C alpha (*PRKCA*) | Downregulated | 4.8E-02 | 0.47 |
| pituitary tumor-transforming 1 (*PTTG1*) | Upregulated | 2.5E-03 | 0.14 |
| zinc finger protein 274 (*ZNF274*) | Upregulated | 4.0E-02 | 0.44 |
| ZW10 interacting kinetochore protein (*ZWINT*) | Upregulated | 1.7E-04 | 0.03 |

p values were adjusted using Benjamini-Hochberg method and expressed as q value.

Supplementary Table S7. NAD biosynthetic process-related genes- GO term Biological Process of ME2 gene module, co-cultured THP-1 macrophages.

| **Genes** | **Regulation** | **p value** | **q value** |
| --- | --- | --- | --- |
| nicotinamide phosphoribosyltransferase (*NAMPT*) | Downregulated | 6.9E-03 | 0.21 |
| NAD(P)HX dehydratase (*NAXD*) | Upregulated | 1.6E-03 | 0.11 |
| poly (ADP-ribose) polymerase family member 16 (*PARP16*) | Downregulated | 5.0E-02 | 0.47 |
| phosphofructokinase, liver type (*PFKL*) | Upregulated | 9.8E-03 | 0.25 |
| phosphofructokinase, platelet (*PFKP*) | Upregulated | 4.2E-02 | 0.21 |

p values were adjusted using Benjamini-Hochberg method and expressed as q value.

Supplementary Table S8. GO Terms-Cellular components of ME2 module, co-cultured THP-1 macrophages.

| Gene set | Description | Size | Expected | Observed | Ratio | P value | FDR |
| --- | --- | --- | --- | --- | --- | --- | --- |
| GO:0005694 | chromosome | 1014 | 8.87 | 26 | 2.93 | 7.2E-07 | 1.7E-04 |
| GO:0015630 | microtubule cytoskeleton | 1165 | 10.20 | 22 | 2.16 | 5.2E-04 | 2.8E-02 |
| GO:0016234 | inclusion body | 79 | 0.69 | 5 | 7.23 | 6.4E-04 | 3.3E-02 |
| GO:0044433 | cytoplasmic vesicle part | 1462 | 12.80 | 25 | 1.95 | 9.0E-04 | 4.1E-02 |
| GO:1990234 | transferase complex | 766 | 6.70 | 16 | 2.39 | 1.1E-03 | 4.9E-02 |

Supplementary Table S9. ANOVA for expression levels of genes categorized for KEGG’s base excision repair (BER), homologous recombination (HR) and non-homologous end joining (NHEJ) pathway.

| Genes involved in base excision repair | Involved KEGG pathway | Regulation | Module eigengene | p value for ANOVA | Mean±S.D. of normalized values  fold change (p value for Dunnett’s multiple comparison) | | |
| --- | --- | --- | --- | --- | --- | --- | --- |
|  |  |  |  |  | 1,2-DCP concentration | | |
|  |  |  |  |  | 0 | 0.1 | 0.4 mM |
| DNA ligase 1 (*LIG1*) | BER | Upregulated | ME9 | 0.0026 | 157±13  1 | 175±15  1.12 (0.16) | 213.0±3.4  1.36 (0.0017) |
| Poly (ADP-ribose) polymerase family member 4 (*PARP4*) | BER | Upregulated | ME9 | 0.037 | 199±15  1 | 192±30  0.96 (0.95) | 271±42  1.36 (0.051) |
| DNA polymerase delta 1, catalytic subuinit (*POLD1*) | BER/HR | Upregulated | ME9 | 0.039 | 128±19  1 | 127±11  0.99 (0.99) | 168±19  1.31 (0.047) |
| 8-Oxoguanine DNA glycosylase (*OGG1*) | BER | Upregulated | ME7 | 0.013 | 148±21  1 | 202±10  1.36 (0.0093) | 187±13  1.26 (0.037) |
| Neil like DNA glycosylase (*NEIL2*) | BER | - | ME7 | 0.19 | 33.5±4.1  1 | 56±15  0.96 | 48±10  1.36 |
| Breast cancer 2 (*BRCA2*) | HR | - | ME6 | 0.024 | 63.7±9.0  1 | 39.2±8.2  0.61 (0.05) | 71±14  1.11 (0.65) |
| Nibrin (*NBN*) | HR | Upregulated | ME8 | 0.043 | 184±21  1 | 195±18  1.06 (0.74) | 236±22  1.28 (0.034) |
| Replication protein A1 (*RPA1*) | HR | Upregulated | ME8 | 0.0029 | 578±43 | 552±47  0.95 (0.71) | 757±47  1.31 (0.0053) |
| Protein kinase, DNA-activated, catalytic subunit (*PRKDC*) | NHEJ | - | ME5 | 0.19 | 151±59  1 | 107±31  0.71 | 176±21  1.17 |

Normalized values of expression level were compared between three groups of different 1-DCP concentration by one-way analysis of variance (ANOVA), being followed by *post hoc* Dunnett’s multiple comparison with control (0mM 1,2-DCP group).
